# The Effects of a 6-Week Strength Training on Critical Velocity, Anaerobic Running Distance, 30-M Sprint and Yo-Yo Intermittent Running Test Performances in Male Soccer Players

**DOI:** 10.1371/journal.pone.0151448

**Published:** 2016-03-25

**Authors:** Bettina Karsten, Eneko Larumbe-Zabala, Gokhan Kandemir, Tahir Hazir, Andreas Klose, Fernando Naclerio

**Affiliations:** 1 University of Greenwich, Department of Life and Sports Science, Kent, England; 2 Texas Tech University Health Science Center, Clinical Research Institute, Lubbock, Texas, United States of America; 3 Hacettepe University, Faculty of Sports Sciences, Ankara, Turkey; 4 Westfälische Wilhelms-Universität Münster, Arbeitsbereich für Sportpädagogik, Münster, Germany; University Hospital of Berne, SWITZERLAND

## Abstract

The objectives of this study were to examine the effects of a moderate intensity strength training on changes in critical velocity (CV), anaerobic running distance (*D*'), sprint performance and Yo-Yo intermittent running test (Yo-Yo IR1) performances. Methods: two recreational soccer teams were divided in a soccer training only group (SO; n = 13) and a strength and soccer training group (ST; n = 13). Both groups were tested for values of CV, *D*', Yo-Yo IR1 distance and 30-m sprint time on two separate occasions (pre and post intervention). The ST group performed a concurrent 6-week upper and lower body strength and soccer training, whilst the SO group performed a soccer only training. Results: after the re-test of all variables, the ST demonstrated significant improvements for both, YoYo IR1 distance (p = 0.002) and CV values (p<0.001) with no significant changes in the SO group. 30-m sprint performance were slightly improved in the ST group with significantly decreased performance times identified in the SO group (p<0.001). Values for *D*' were slightly reduced in both groups (ST -44.5 m, 95% CI = -90.6 to 1.6; SO -42.6 m, 95% CI = -88.7 to 3.5). Conclusions: combining a 6-week moderate strength training with soccer training significantly improves CV, Yo-Yo IR1 whilst moderately improving 30-m sprint performances in non-previously resistance trained male soccer players. Critical Velocity can be recommended to coaches as an additional valid testing tool in soccer.

## Introduction

One of the most important variables for measuring performance in soccer is physical conditioning [1]. A significant relationship between the maximal aerobic conditioning of a player and both, the number of sprints attempted and the distance covered during a game has been established [2].

Critical to the success in soccer are strength and power actions [3,4]. These have been demonstrated to develop through specific training and are intrinsically linked to improved overall match performances [5–7]. The performance levels of such actions expressed as maximum strength can distinguish between competitive levels of soccer players and a strong relationship between maximal strength and sprint times has been suggested [8].

Another important factor of soccer specific skills is power with the two main components of velocity and strength and recent researchers demonstrated the positive effects of a strength training (ST) on indices of performance [9–11]. However, to-date there is only limited evidence, which suggests similar results for soccer. Helgerud et al. [12] demonstrated a 51.7% maximal resistance improvement (1-RM) after 8 weeks of half-squat training, which also translated in sprint performance improvements. In rugby, Barr et al. [13] emphasized a strong relationship between lower-body strength and sprint speed. Moreover a 3-week maximal ST has been recommended as an effective method to improve sprint and aerobic performances in soccer [14].

Soccer coaches commonly use the Yo-Yo intermittent recovery test (Yo-Yo IR1) to monitor chances in aerobic performance capacities. Whilst Critical Velocity (CV) testing is not typically performed, it has been suggested as a worthwhile and easily accessible additional testing tool [15]. For an intermittent sport such as rugby, the same researchers [15] demonstrated CV to be an appropriate test which reflects the highest rate of aerobic metabolism without a progressive loss in metabolic steady-state. CV once exceeded results in the utilization of the anaerobic running distance (*D*'; expressed in meters). CV furthermore demarcates the boundary between the heavy and the severe exercise intensity [16]. Importantly a CV and *D*' enhancing training is beneficial for players whilst the values of CV and *D*' can be utilized as an easy tool to monitor changes in performance capacities as well as aid in the prescription of training [17] Whilst the tests of CV/*D*' and Yo-Yo IR1 might differ in variation of energetics used and in character of locomotion, i.e. continuous versus intermittent, the relationship between these has only been investigated in women’s rugby [15]. The study identified a high correlation (r = 0.86) between CV and YoYo IR1 results.

To-date the limited evidence on the effects of ST on CV has only been analyzed in continuous-cyclic sports. In cycling Bishop and Jenkins [18] demonstrated a positive, albeit a non-significant, impact of such intervention on Critical Power (the analogous of CV) and a significant increase in *W*' (the analogous of *D*'). Supporting this Sawyer et al. [19] also demonstrated a non-significant change in CP whilst significant improving *W*' and time-to-exhaustion durations after an 8-weeks ST intervention. More recently, Karsten et al. [20] found a non-significant but meaningful 3% improvement for CV with a significant decrease in *D*' in recreational runners. Research that addresses the impact of ST on intermittent sports, measured in changes of CV and *D*' has yet to be undertaken.

The objective of the study was therefore to investigate the effectiveness of a ST intervention on Yo-Yo IR1 results, 30-m spring performance and CV and *D*' in soccer players. Additionally the relationships between test results for the Yo-Yo IR1 and CV and for the 30-m sprint performance and *D*' were investigated. Based on previous findings an improvement in all variables but *D*' and a strong relation between CV and Yo-Yo IR1 results was hypothesised.

## Methods

### Subjects

**Fig 1:**
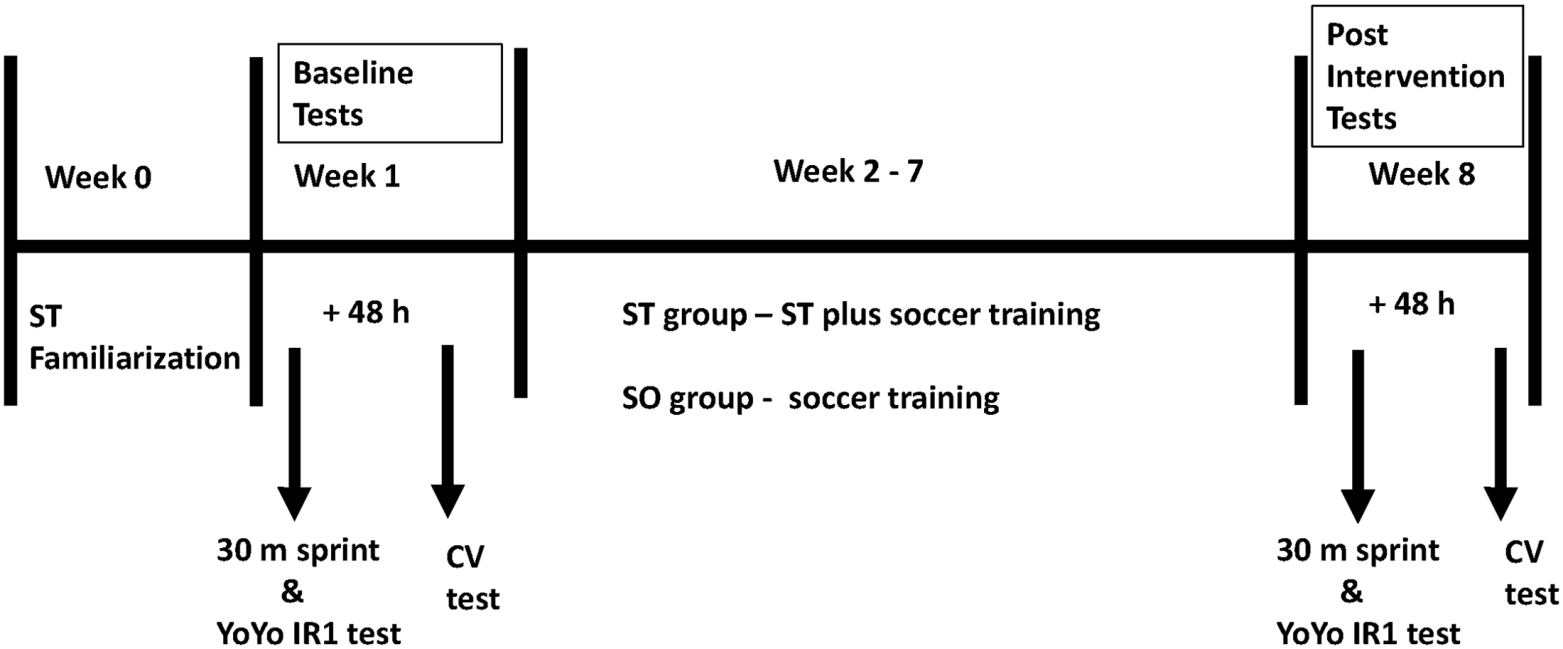
Testing timeline: testing period at baseline and after the intervention. ST = experimental group (performing ST + soccer training from week 2–7; SO = soccer training group (soccer training from week 2–7).

Twenty-six recreational field soccer players from two Turkish Soccer Federation Third League teams participated in the present study which took place during the preparatory period of the soccer season (Fig 1). All players were regularly involved in competitive soccer games and had been training for a minimum of two years. At the time of the investigation players were performing 3 weekly training sessions containing low intensity aerobic training (i.e. 70–85 HRmax), plyometric, agility and soccer specific drills with no ST as integrated as part of physical conditioning program. Players were not familiar with maximal ST.

The teams were divided into an experimental strength and soccer training (ST, n = 13: 18 ± 1 yr., height 174.8 ± 5.4 cm, body mass 72.4 ± 6.6 kg) and a soccer only training group (SO, n = 13: 19 ± 1 yr., height 177.3 ± 5.2 cm, body mass 73.2 ± 7 kg).

Subjects agreed to refrain from intense exercise and alcohol consumption on the day preceding any tests and not to consume a major meal or caffeine 3 hours prior testing. In addition subjects were not allowed to perform any other exercises than those required in the partaking of the investigation. All subjects were fully informed about the study procedure, protocols, benefits and risks. A health history questionnaire was used to ensure subjects were healthy and free of any musculoskeletal injury or cardiovascular disease. The study was carried out in accordance with the guidelines contained in the Declaration of Helsinki and was approved by the faculty of sports science ethic committee of Hacettepe University, Turkey. Participants had to provide a full written consent prior to testing.

### Procedures

This study utilized a two parallel-group controlled design, where two between-participant conditions ST and SO were tested. Subjects were instructed to maintain a similar soccer training throughout the experimental period. Prior to the start of the study, the ST group completed a familiarization period for all resistance training exercises. This was followed by two testing sessions, where CV/*D*' (test 1) and the 30-m sprint test followed by the Yo-Yo IR1 (test 2) were performed. All testing sessions were carried out with a 48 h recovery period. Players were familiar with the Yo-Yo IR1 test and had performed the test prior to the present study at least once. Consequently the ST group performed on separate days a twice-weekly ST training in addition to the thrice-weekly soccer training and the SO group continued with their regular thrice-weekly soccer training. After the experimental period of 6 weeks, both testing sessions were repeated (Fig 1).

### Measurements

#### 30-m sprint test and Yo-Yo IR1 test

After a standardized warm-up (5-min self-paced jogging followed by a 5-min stretching routine), from a standing start, players performed three repeated 30-m sprints using a 60 s recovery between sprints outdoor on a football field. Times were recorded using timing gates and the best individual sprint time was used for data analysis. This was followed by the Yo-Yo IR1 test where 3–4 players performed the test together. Consistent with Bangsbo, Iaia and Krustrup [21] players had to run repeatedly 20-m runs back and forth between the starting, turning and finishing line on the outdoor soccer field. Starting a 10 km·h^-1^, the pace at which players had to run progressively increased throughout the test and followed audio bleep clues played from a CD player. Between each bout of running, players had a 10-s active resting period, which consisted of 2 times 5 m jogging pace. The testing distance was marked by cones, having a width of 2 m and a length of 20 m. Another cone placed 5 m behind the finishing which marked the running distance during the active recovery period. Tests were terminated when players twice failed to reach the finish line in time. Distance covered was recorded to the nearest meter. All players were familiarized to the test by one pre-test.

#### CV tests

Subjects had to run 3000 m, 1800 m and 1000 m in a fastest possible time around an athletic track, using a 30 minutes recovery method between trials [22]. Runs were conducted in this order and repeated after the experimental period (7.5, 4.5 and 2.5 laps). Distances chosen attempted to induce exhaustive runs between 2 and 15 min. Trials were performed individually and used the standardized warm-up (5-min self-paced jogging followed by a 5-min stretching routine). Players were strongly verbally encouraged throughout each trial and time was recorded using timing-gates. A 5-minute cool-down at self-paced speeds were performed before passively resting for another 25 minutes. Testing was not performed if wind speed exceeded 2 m·s^-1^. Linear regression was used to determine CV and *D*' (SE = 0.15 m·s^-1^) using the distance—time relationship [(d = CV * t) + *D*'], where: d = distance run and t = total running time.

#### Strength Training

The ST group performed a 6-week ST program involving three-resistance lower body (parallel squat, lunges, romanian deadlift) and two-resistance upper body (dumbbell upright row and bench press) exercises. The ST program was performed twice-weekly on non-consecutive days (12 sessions in total). In order to determine the training load of each of the selected exercise, subjects included in ST group performed a maximal strength test (1-RM) for all five selected exercises. The 1-RM value for each of the 5 resistance exercises was determined according to the methodology proposed by Baechle, Earle and Wathen [23]. During the first two weeks, players performed 3 sets of 12 repetitions at 60–65% 1RM for each exercise at. During the following 4 weeks subjects performed 3 to 4 sets of 70–75% 1RM for all exercises. Loads were adjusted based on the rate of perceived exertion [RPE OMNI-Res Scale; [24]]. When subjects expressed a value lower than 9 at the end of the last set, load was increased between 5% to a maximum of 15%, depending on the players feedback. Subjects were instructed to perform exercises as fast as possible using a proper technique. A certified Strength and Conditioning Coach supervised all ST workouts.

### Statistical Analysis

A descriptive analysis was performed and subsequently the Kolmogorov-Smirnov and Shapiro-Wilk test were applied to assess normality. The same mixed Analysis of Variance (ANOVA) model was applied to test the differences in each dependent variable (CV, *D*', YoYo IR1, and 30 m sprint) compared by group (ST vs. SO) and over time (pre vs. post). Pairwise comparisons were used to determine differences between time points. Generalized eta squared ($\eta_{G}^{2}$) and Cohen’s *d* values were reported to provide an estimate of standardized effect size (small d = 0.2, $\eta_{G}^{2}$ = 0.01; moderate d = 0.5, $\eta_{G}^{2}$ = 0.06; and large d = 0.8, $\eta_{G}^{2}$ = 0.14). The significance level was set to p*<*0.05. Results are reported as mean ± SD unless stated otherwise. IBM SPSS statistics version 20.0 package was used to perform descriptive statistics and ANOVA. Effect sizes were calculated by hand based upon Bakeman [25]. Relationships were assessed using Pearson product moment correlation coefficients.

## Results

All data were normally distributed. Average running times for the 3000 m were 13.95 ± 1.37 min, for the 1500 they were 7.26 ±0.6 min and for the 1000 m they were 3.53 ± 0.47 min. Table 1 shows the values measured for CV, *D*', YO-YO IR1 and sprints for the experimental and the control group. When converting the YoYo IR1 from distance run (m) into final speed (m/s) a mean moderate relationship (r = 0.31) between the variables was identified (Table 1).

**Table 1:** Baseline and post intervention values for YoYo IR1, CV, *D'* and 30-m sprint performances (±SD) for the experimental and control group (*P* < 0.05).

	ST (n = 13)		SO (n = 13)	
	Pre	Post	Pre	Post
Yo-Yo IR1 (m)	1547.7 ± 315.1	1695.4 ± 307*	1427.7 ± 368.2	1430.8 ± 309.5
Yo-Yo IR1 (m/s)	4.44 ± 0.2	4.48 ± 0.1	4.38 ± 0.2	4.39 ± 0.1
CV (m/s)	3.43 ± 0.29	3.75 ± 0.23*	3.23 ± 0.34	3.35 ± 0.27
*D'* (m)	300.8 ± 64.8	256.3± 39.2	337.1 ± 80.7	294.5 ± 111.8
30-m (s)	4.68 ± 0.2^¥^	4.56 ± 0.24	4.53 ± 0.14	4.56 ± 0.22**

* Significantly improved pre and post test results;

**Significantly decreased pre and post test results;

^¥^ Significantly different between groups

### YoYo IR1 test

The ANOVA results showed a significant interaction effect between group and time, F(1,24) = 11.22, p = 0.003, $\eta_{G}^{2}$ = 0.01. Pre to post differences were significant (F(1,24) = 12.2, p = 0.002, $\eta_{G}^{2}$ = 0.01) but we did not find statistically significant group effect (F(1,24) = 2.33, p = 0.140, $\eta_{G}^{2}$ = 0.09). Although the SO group showed a non-significant improvement (95% CI = -59.9 to 66.1 m/s, t(24) = 0.1, p = 0.921, d = 0.02), the ST group covered 147.7 m more (95% C.I. = 84.7 to 210.7) at the YoYo IR1 test after completing the specific training, t(24) = 4.84, p<0.001, d = 0.95. Using the predictive maximal aerobic capacity (VO_2max_) equation for the Yo-Yo IR1 test (VO_2max_ = IR1 distance (m) × 0.0084 + 36.4) the prior and post intervention values for the ST group were 49.4 ± 2.6 mL·min·kg and 50.6 ± 2.6 mL·min·kg and for the SO group they were 48.4 ± 3.1 mL·min·kg and 48.4 ± 2.6 mL·min·kg. In order to determine the statistical power, a post-hoc analysis was conducted of the primary outcome (Yo-Yo IR1) for the ST group (n = 13, d = 0.95). Assuming a significance level of 0.05 we reached a power of 0.88.

### Critical velocity

Significant differences by group (F(1,24) = 9.81, p = 0.005, $\eta_{G}^{2}$ = 0.23) and time (F(1,24) = 15.48, p = 0.001, $\eta_{G}^{2}$ = 0.11) were found. The interaction effect was not significant (F(1,24) = 3.34, p = 0.080, $\eta_{G}^{2}$ = 0.03). The SO players’ changes in CV after six weeks was not significant (95% CI = -0.16 to 0.48 m/s, t(24) = 1.48, p = 0.152, d = 0.29). In contrast, ST players improved CV by 0.32 m/s (95% C.I. = 0.16 to 0.48) after completing the specific training, t(24) = 4.05, p<0.001, d = 0.79.

### Anaerobic running distance

Only the pre-post effect was found to be significant (F(1,24) = 7.59, p = 0.011, $\eta_{G}^{2}$ = 0.07), but no significant difference between groups (F(1,24) = 1.98, p = 0.172, $\eta_{G}^{2}$ = 0.06) or interaction effects (F(1,24) = 0.003, p = 0.953, $\eta_{G}^{2}$ ≈0) were found. Anaerobic running distance was slightly reduced in both groups (ST -44.5 m, 95% CI = -90.6 to 1.6; SO -42.6 m, 95% CI = -88.7 to 3.5), showing similar trends in the ST group (t(24) = -1.99, p = 0.058, d = -0.39) and SO group (t(24) = -1.91, p = 0.069, d = -0.37).

### 30-m Sprint test

The ANOVA results showed a significant interaction effect between group and time, F(1,24) = 88.78, p<0.001, $\eta_{G}^{2}$ = 0.28. Pre to post effect (F(1,24) = 144.14, p<0.001, $\eta_{G}^{2}$ = 0.31) group effect (F(1,24) = 75.82, p<0.001, $\eta_{G}^{2}$ = 0.49) were also significant. SO players worsened their performance at the sprint test (t(24) = 15.25, p<0.001, d = 2.99) by about 0.98 m/s (95% CI = -1.11 to -0.84). In contrast, no significant differences were observed for the ST group (t(24) = 1.84, p = 0.078, d = 0.36; 0.12 m/s, 95% CI = -0.25 to 0.15) with some players (n = 11) demonstrating improved sprint performances.

## Discussion

The main findings of the present study were that a 6-week, twice-weekly performed ST training program resulted in a significant increase in YoYo IR1 and CV performances in male soccer players (Table 1). Values for 30-m sprint performances decreased by a mean non-significant 0.12 ± 0.14 s in the experimental group only, whilst values of *D*' significantly decreased by a mean of 45 ± 82 m and 43 ± 80 m in both, the experimental and the control group respectively. When converting the Yo-Yo IR1 distance results into running velocity, the mean performance changes expressed as a percentage do not reflect the overall improvements, when compared to changes in distance covered and CV values in both groups (ST 0.75 ± 1.9%; SO 1.23 ± 1.62% vs ST 9.8 ± 3.7 m/s; SO 4.23 ± 8.86 m/s for YoYoIR1 and CV values respectively). This suggests that Yo-Yo IR1test running velocities are less sensitive to changes in performance capacity.

Our findings are supported by Karsten et al. [20] who reported a 2.9% increase in CV after a 6-week ST intervention in moderately training runners. The present study resulted in markedly larger significant improvements (Table 1; 9.8%), which might have been caused by different training levels or by the differences in ST exercises as Karsten et al. [20] only applied lower body exercises. Whilst not part of the present investigation Støren et al. [26] demonstrated improvements in running economy after an implementation of upper-body maximal ST, which can be suggested to have influenced the comparatively higher changes in CV values in the ST group. In contrast to our findings Bishop and Jenkins [18] and Sawyer et al. [19] reported non-significant changes in CP after a ST intervention using 6-weeks and 8-weeks respectively. Bishop and Jenkins [18] implemented only lower-body ST exercises whist Sawyer et al. [19] included some upper-body ST exercises. Unlike the present study both aforementioned interventions were performed using untrained subjects. Moreover, the differences between continuous and intermittent activities, such as change intensities, change of directions and acceleration and deceleration, might account for the divergent findings.

Deemed as a suitable fitness test for an intermittent sport such as rugby [15] results of the present study demonstrate that CV testing can also be applied in soccer. However, the present study only identified a moderate correlation between CV and Yo-Yo IR1 tests results. Unlike the present study, Clarke et al. [15] utilised running distances of 100 m, 400 m and 1500 m for the determination of CV which resulted in shorter mean running times (13.8 s, 1.24 min and 7.85 min respectively). Poole et al. [16] recommended a minimum exhaustive trial duration of 2 minutes which might explain the discrepancies between findings, as shorter exhaustive trials produce higher CV values [27].

The Yo-Yo IR1 has demonstrated its reliability and ability to discriminate between several age groups as well as playing positions [28–30]. Our novel findings suggest an equal testing sensitivity of CV, as both variables improved by the same magnitude (YoYo IR1 [m]) 10.2%; CV [m/s] 9.8%). CV may therefore offer an additional valid and ecologically enhanced useful testing tool as athletes are not required to perform a systematically increase in running velocity to exhaustion, a physiological stress that is only limited reflective of the game of soccer. Moreover CV can be used as a non-invasive method to determine the lactate turnpoint [31] as well as it provides an objective, valid, reliable, accurate and sensitive testing method [32] to monitor changes in endurance fitness [33]. It can furthermore be used as a reference value to determine the boundary between the heavy and severe endurance training zone [16].

It is widely accepted that the implementation of ST improves sprint performances at 10–40 m [34] and our findings of a mean 2.6 ± 0.02% albeit non-significant sprint performance increase are supported by several investigations. [12] demonstrated a significant 1.6% sprint performance improvement over a distance of 20 m using a half-squat exercise in elite soccer players after 8 weeks (twice weekly training). Ronnestad et al. [6] in professional soccer players found an increase of 1.3% in 40-m sprint performance after a 7-week intervention (twice weekly training) of half-squat and hip flexor exercises. The higher magnitude improvement in sprint performance in the present study may be either due to the number of relevant ST exercises or the level of players as the duration, volume and intensity of the interventions are similar. Kotzamanidis et al. [35] however reported conflicting 30-m results as players demonstrated only small improvements in sprint performance (0.46%). The training consisted of half-squat, leg curl and step-up exercises over a 9-week period using a periodized heavy RT. The authors did not report the level of players, which may explain the different findings.

Our study also identified notable decreases in *D*' in both groups (Table 1). Consistent with Karsten et al. [20] who reported a decrease for this parameter after a 6-weeks ST intervention in moderately trained runners our findings are in contrast to those of Bishop and Jenkins [18] and Sawyer et al. [19]. These conflicting results support a current disagreement in the literature on the reliability, true constitution and adaptive behaviour of this parameter of the time-distance relationship [36–39]

### Limitation to the study

The limited access to players did not allow for a post intervention 1-RM test of the ST exercises. The study design furthermore does not allow us to definitive state, whether the presented improvements are solely due to the ST treatment, as training loads were not equalised between groups.

## Conclusions

To significantly improve performance capacities, a 6-week concurrent soccer and ST program using moderate (60–65% 1 RM) to heavy (70–75% 1RM) loads can be recommended to soccer players with no experience in resistance training during the preparatory period of the soccer season. Determining the transition between heavy and severe exercise and exercise tolerance, the present study also demonstrated the usefulness of CV as a tool to detect changes in performance capacities induced by the ST intervention. CV can therefore be recommended to soccer coaches as additional suitable, valid, sensitive and reliable test, which can additionally be used for the purpose of training prescription. Future studies are recommended which equalize training loads, to investigate the optimal duration and hence an optimal performance improvement of such sports specific ST training.

## Supporting Information

S1 Table Supporting data set. (PDF)

S1 Fig Flow diagram. (PDF)
