# Supplementary material for: The Effects of a 6-Week Strength Training on Critical Velocity, Anaerobic Running Distance, 30-M Sprint and Yo-Yo Intermittent Running Test Performances in Male Soccer Players
Source: PLoS One. 2016 Mar 25;11(3):e0151448. doi: 10.1371/journal.pone.0151448 (PMC4807759; doi:10.1371/journal.pone.0151448)
Supplement: S1 Table — (PDF) [file pone.0151448.s001.pdf]

Group 1 = Exp; 2 = Control	Pre Yoyo	Post Yoyo
1	1720	1880
1	1200	1360
1	1200	1400
1	1320	1480
1	1480	1560
1	1920	2040
1	1800	1960
1	1040	1200
1	1800	1720
1	2120	2320
1	1440	1640
1	1520	1760
1	1560	1720
2	1320	1240
2	2160	2040
2	1240	1320
2	1960	1800
2	1520	1400
2	1400	1480
2	1600	1480
2	640	840
2	1360	1520
2	1280	1160
2	1480	1560
2	1440	1640
2	1160	1120

Pre CV	Post CV	Pre D'	Post D'
3.32	4.03	355.39	232.35
3.75	3.90	217.75	225.44
3.26	3.41	214.44	318.39
3.54	3.81	320.03	253.63
3.42	3.65	320.27	264.67
4.08	4.13	205.57	215.90
3.24	3.83	373.83	251.50
3.36	3.70	302.14	241.10
3.33	3.44	366.10	316.68
3.16	4.02	357.19	246.10
3.06	3.68	340.09	210.20
3.29	3.67	327.07	231.76
3.83	3.53	210.35	324.05
2.80	2.82	453.18	450.82
3.51	3.32	263.38	293.49
3.57	3.78	300.91	262.16
3.23	3.06	393.28	398.66
3.48	3.58	289.54	146.45
2.79	3.25	471.86	367.78
3.50	3.70	180.35	103.30
2.65	3.35	379.83	145.75
3.59	3.56	311.80	320.49
2.88	3.08	297.81	241.66
3.12	3.24	384.10	421.12
3.50	3.46	369.24	384.98
3.38	3.32	287.58	291.97

Pre 30 M	Post 30 M
4.53	4.3
4.84	4.71
4.4	4.4
4.7	4.59
4.56	4.55
4.68	4.67
5.18	5.16
4.6	4.3
4.73	4.69
4.89	4.52
4.63	4.62
4.52	4.55
4.52	4.19
4.59	3.11
4.75	3.59
4.52	3.92
4.45	3.4
4.5	3.58
4.43	3.52
4.47	3.69
4.78	3.47
4.3	3.8
4.62	3.27
4.63	3.62
4.39	3.72
4.48	3.53
