# Supplementary figures and images for: The Effects of a 6-Week Strength Training on Critical Velocity, Anaerobic Running Distance, 30-M Sprint and Yo-Yo Intermittent Running Test Performances in Male Soccer Players
Source: PLoS One. 2016 Mar 25;11(3):e0151448. doi: 10.1371/journal.pone.0151448 (PMC4807759; doi:10.1371/journal.pone.0151448)

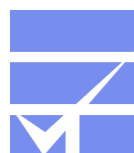

# CONSORT

TRANSPARENT REPORTING of TRIALS

## CONSORT 2010 Flow Diagram

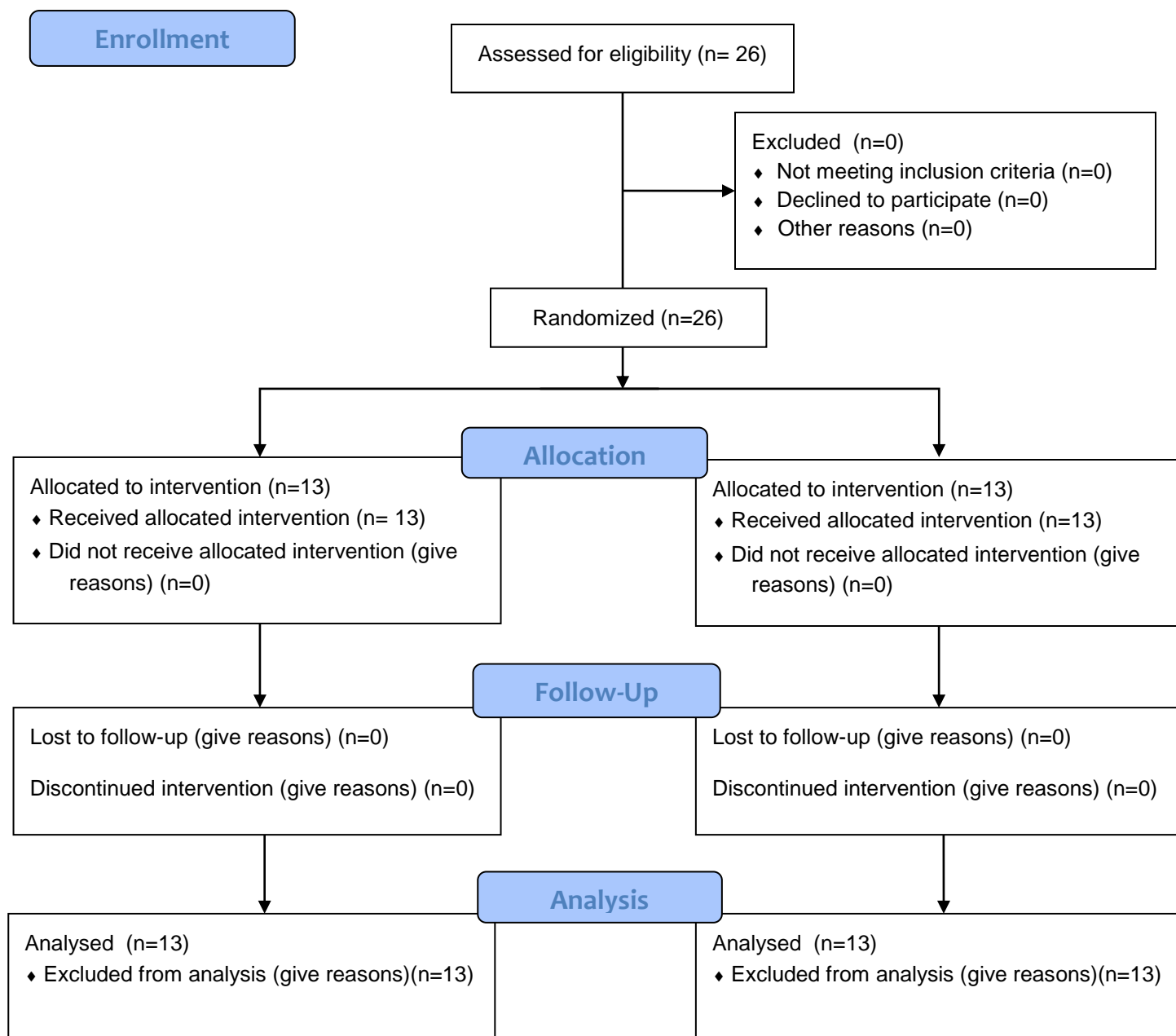

Supplement: S1 Fig — (PDF) [file pone.0151448.s002.pdf]
